# The rapid detection of respiratory pathogens in critically ill children

**DOI:** 10.1186/s13054-023-04303-1

**Published:** 2023-01-10

**Authors:** John A. Clark, Andrew Conway Morris, Martin D. Curran, Deborah White, Esther Daubney, Iain R. L. Kean, Vilas Navapurkar, Josefin Bartholdson Scott, Mailis Maes, Rachel Bousfield, M. Estée Török, David Inwald, Zhenguang Zhang, Shruti Agrawal, Constantinos Kanaris, Fahad Khokhar, Theodore Gouliouris, Stephen Baker, Nazima Pathan

**Affiliations:** 1 Department of Paediatrics, Addenbrooke’s Hospital, University of Cambridge, Level 8, Cambridge Biomedical Campus, Cambridge, CB2 0QQ UK; 2 Cambridge University Hospitals NHS Foundation Trust, Cambridge, UK; 3 Division of Anaesthesia, Department of Medicine, University of Cambridge, Cambridge, UK; 4 Division of Immunology, Department of Pathology, University of Cambridge, Cambridge, UK; 5 Clinical Microbiology and Public Health Laboratory, United Kingdom Health Security Agency, Cambridge, UK; 6 Cambridge Institute of Therapeutic Immunology and Infectious Disease, University of Cambridge, Cambridge, UK; 7 Division of Infectious Diseases, Department of Medicine, University of Cambridge, Cambridge, UK; 8 Blizard Institute, Queen Mary University of London, London, UK

**Keywords:** Pneumonia, Critical care, Paediatric, Diagnostic techniques, Respiratory system, Healthcare-associated pneumonia

## Abstract

**Purpose:**

Respiratory infections are the most common reason for admission to paediatric intensive care units (PICU). Most patients with lower respiratory tract infection (LRTI) receive broad-spectrum antimicrobials, despite low rates of bacterial culture confirmation. Here, we evaluated a molecular diagnostic test for LRTI to inform the better use of antimicrobials.

**Methods:**

The Rapid Assay for Sick Children with Acute Lung infection Study was a single-centre, prospective, observational cohort study of mechanically ventilated children (> 37/40 weeks corrected gestation to 18 years) with suspected community acquired or ventilator-associated LRTI. We evaluated the use of a 52-pathogen custom TaqMan Array Card (TAC) to identify pathogens in non-bronchoscopic bronchoalveolar lavage (mini-BAL) samples. TAC results were compared to routine microbiology testing. Primary study outcomes were sensitivity and specificity of TAC, and time to result.

**Results:**

We enrolled 100 patients, all of whom were tested with TAC and 91 of whom had matching culture samples. TAC had a sensitivity of 89.5% (95% confidence interval (CI_95_) 66.9–98.7) and specificity of 97.9% (CI_95_ 97.2–98.5) compared to routine bacterial and fungal culture. TAC took a median 25.8 h (IQR 9.1–29.8 h) from sample collection to result. Culture was significantly slower: median 110.4 h (IQR 85.2–141.6 h) for a positive result and median 69.4 h (IQR 52.8–78.6) for a negative result.

**Conclusions:**

TAC is a reliable and rapid adjunct diagnostic approach for LRTI in critically ill children, with the potential to aid early rationalisation of antimicrobial therapy.

**Supplementary Information:**

The online version contains supplementary material available at 10.1186/s13054-023-04303-1.

## Introduction

Respiratory tract infections are the leading cause of admission onto paediatric intensive care units (PICUs). In 2019, 6174 were children admitted to UK PICUs with a primary respiratory illness [1]. At a cost of ≥ £3,713 per occupied bed day in a PICU, these cases constitute a significant burden to the National Health Service (NHS) [2]. With such severe disease presentations, PICU clinicians generally initiate early broad-spectrum antimicrobial therapy [3], which may affect routine microbiological culture results [4, 5]. Most children are likely to have viral infections, but two factors are key in the de-escalation of antimicrobial therapy in this cohort—lack of rapid diagnostic tests to determine bacterial co-infection and a low prevalence of positive respiratory cultures.

Quantitative PCR (qPCR) has been used for many years to identify respiratory viruses in nasopharyngeal swabs or aspirates for children. A recent study of critically ill adults with suspected lower respiratory tract infection (LRTI) found that the use of a qPCR-based rapid pathogen array reduced the use of inappropriate antimicrobial therapy compared to standard diagnostic microbiology [6]. The potential for respiratory pathogen qPCR arrays to detect bacterial and fungal respiratory pathogens in hospitalised children has been discussed, but the outcome of their clinical implementation is yet to be reported [7, 8].

The TaqMan Array Card (TAC) (Thermo Fisher Scientific, California) [9] is a microfluidic qPCR system which is comprised of 384 wells containing prealiquoted customised primer and probe combinations [9]. We have previously reported the use of a custom TAC to support ventilator-associated pneumonia (VAP) diagnosis in adults [10, 11]. Here we sought to assess the utility of this TAC to identify bacterial and fungal respiratory pathogens in critically ill children with suspected community acquired pneumonia (CAP) or VAP.

The primary objectives of this study were to determine the sensitivity and specificity of TAC to detect bacterial and fungal pathogens causing LRTI, and to compare time to result of TAC versus standard microbiology cultures. The secondary objectives of the study were to describe the micro-organisms detected by TAC that were not detected using microbiology culture; assess the impact of TAC on total antimicrobial prescriptions in the PICU; and describe the impact of TAC on antimicrobial decision-making according to PICU consultants.

## Methods

### Study design

The Rapid Assay for Sick Children with Acute Lung infection Study (RASCALS) was a single-centre prospective observational cohort study of children admitted to PICU conducted in a 13-bed general PICU in the East of England as previously described [12].

### Enrolment and consent procedures

Children ≤ 18 years of age were eligible for inclusion if they were mechanically ventilated and had commenced or were commencing antimicrobial therapy for LRTI. Children aged < 37 weeks corrected gestation were excluded in addition to those predicted to have non-survivable illness. Premature infants were not included given safety, and adequacy of the sampling procedure requires further assessment in this group. Screening for eligibility was carried out by clinical staff based on the defined criteria. Patients were enrolled consecutively on weekdays based on availability of laboratory staff for sample processing. A deferred consent approach was taken for enrolment in the project, given the time-critical nature of investigations. Consent was obtained from carers, and children who had the capacity to give consent. Assent was obtained from children deemed to be Gillick competent.

### Study procedures

**Fig. 1:**
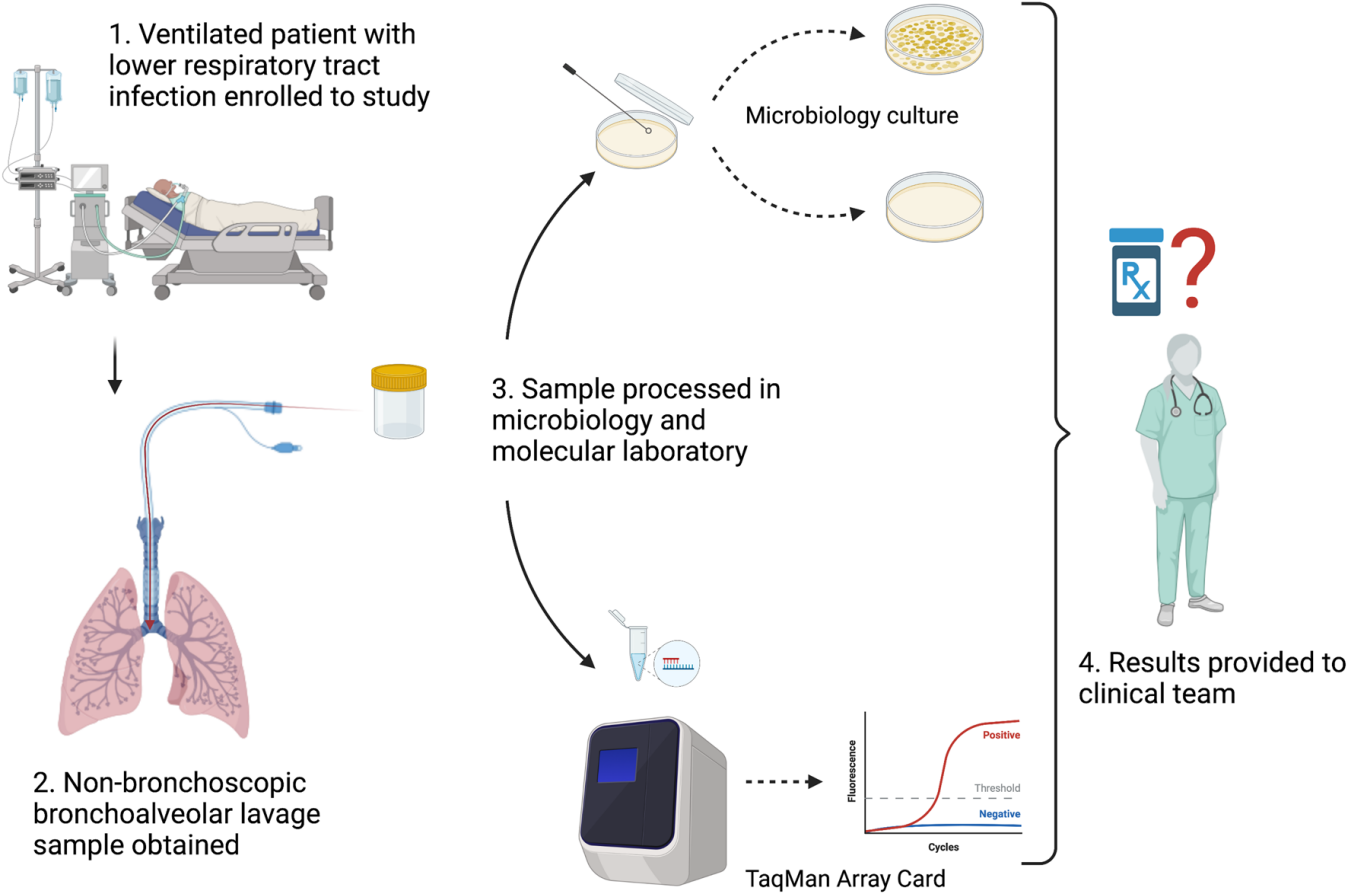
Study workflow. This figure represents the protocol of the Rapid Assay for Sick Children with Acute Lung infection Study. Critically ill children with suspected respiratory infection underwent non-bronchoscopic bronchoalveolar lavage. Specimens that were obtained underwent both routine microbiology culture and custom TaqMan Array Card. Results were reported back to clinicians, and consultants were surveyed regarding their antimicrobial decision-making

Following enrolment, children received a non-bronchoscopic bronchoalveolar lavage (mini-BAL). This procedure involves instillation and re-aspiration of saline (1 mL/kg up to 10 mL) using an in-line suction catheter at the level of the carina. Despite the protocol recommending the mini-BAL method, endotracheal tube (ETT) aspirate samples were still included. Samples were divided in the microbiology laboratory into aliquots for routine microbiological testing in addition to extraction of nucleic acid (Fig. 1). Nucleic acid was extracted from samples using an EZ1 virus mini kit (v2.0) using an EZ1 advanced XL (Qiagen) machine and loaded onto TAC. The TAC was processed on a QuantStudio 7 qPCR machine (ThermoFisher) [12]. Results were reported by an experienced laboratory member and entered into the electronic medical record (EMR) (Epic Systems Corporation, Wisconsin). PICU consultants were surveyed regarding their actions following availability of these results via an email link to the electronic data capture instrument.

### Test interpretation

The TAC incorporated molecular probes targeting species-level bacteria in addition to higher taxonomic groups. Sequences have previously undergone technical validation [10]. Each target was reported according to cycle threshold (Ct), which represents the number of cycles of PCR amplification that occurred before exponential increase in the fluorescent reporter signal; a positive result was inferred if a Ct ≤ 32 was recorded. This threshold was selected based on the median value of detection based on the former study in adults [10]. Cultures were reported as positive if ≥ 10^4^ colony-forming units (CFU)/mL were isolated from mini-BAL samples, and ≥ 10^5^ CFU/mL from ETT aspirate samples. Culture was used as the gold standard for comparison to TAC. Mini-BAL was the recommended sampling approach to avoid contamination from upper respiratory microbiota. Validation of bacterial pathogens was carried out using sequencing of the V3-V4 region of the bacterial 16S rRNA gene (16S sequencing) (Additional file 1).

### Data collection

Data were recorded in an electronic research database, RedCAP, hosted by the University of Cambridge [13]. Demographic information (age at admission, admission source, diagnosis and comorbidities, days on mechanical ventilation, and duration of admission), all routine microbiology and virology investigations, antimicrobial prescription data, and Paediatric Index of Mortality 3 scores [14] were collected. Investigation results and clinical characteristics at the time of mini-BAL sample were also recorded [14]. The duration of PICU stay is reported as days-free-of-PICU 28 days following admission. The duration of treatments are reported as days-free-of-treatment 28 days following admission to PICU. These are standard composite measures that capture the impact of mortality, with days following death considered unliberated from treatment and/or admission for the purposes of the calculations [15].

### Sample size

The sample size was calculated to detect a 60% increase in the sensitivity of TAC to detect bacteria in the samples compared to microbiological culture. A previous investigation on this PICU identified that the prevalence of positive respiratory cultures was 22% [5]. The power calculation determined that 85 participants were required, which was rounded to 100 to account for loss to follow-up and sample failure.

### Statistical analysis

Demographic data were reported with mean and standard deviation, unless the distribution of data was skewed in which case median and interquartile range were used. As there was no ideal ‘gold standard’ test for comparison, TAC and culture results were pooled to determine overall sensitivity and specificity. To determine the sensitivity and specificity of TAC for the detection of bacterial and fungal species, we analysed 25 species on the array which are detectable with the microbiology culture techniques advised for respiratory samples [16]. Genus- or family-level targets were not included in the calculations of diagnostic performance. The detection of bacteria found only by TAC (without a positive culture) underwent secondary validation by 16S sequencing.

The time to reported TAC or microbiology culture result was measured as the time of sample collection to the documented result time in the EMR and compared using the Wilcoxon signed-rank test.

To determine impact of TAC on antimicrobial prescribing, study patients were matched to control patients with suspected LRTI admitted to the same PICU between October 2019 and February 2020 [5]. Demographic features were compared using the Mann–Whitney U test for nonparametric data and the Student’s t-test for normally distributed data. Proportions were compared using the Chi-square test for independence. Antimicrobial use was compared by the Fisher’s exact test. Graphs were generated with R studio v7.1, R version 4.2.0 using ggplot2 [17, 18] and the figures created using Biorender.com.

Antimicrobial use was reported according to the number and percentage of days of the PICU stay in which the patient was exposed to treatment. Exposure to a single dose of an antimicrobial between 00:00 and 23:59 h was one day of exposure. Given that many patients were on multiple courses of antimicrobial therapy, antimicrobial data were also reported according to an antimicrobial spectrum index (ASI). ASI is an approach that allocates a score to systemic antimicrobials according to the range of organisms for which they have therapeutic benefit [19, 20]. The ASI score was multiplied by the number of days of exposure to each antimicrobial in the PICU and summed to estimate total antimicrobial usage. Three antimicrobials used in PICU were not included in the published ASI tools: ceftolozane–tazobactam, flucloxacillin, and teicoplanin; therefore, a score was determined independently for these antimicrobials (Additional file 1: Table S1).

## Results

### Study population

**Fig. 2:**
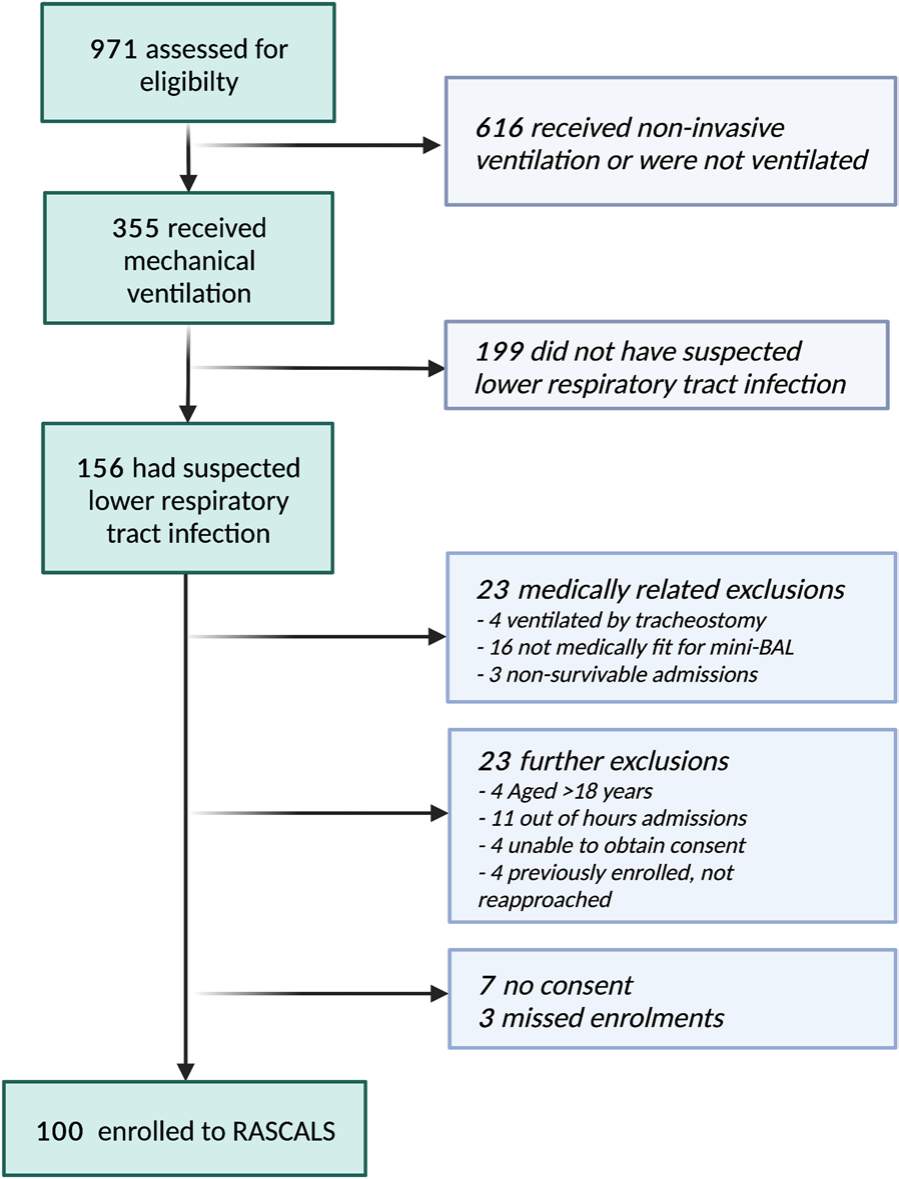
RASCALS enrolment flowchart. Flowchart of children screened and enrolled into the Rapid Assay for Sick Children with Acute Lung infection Study (RASCALS) April 2020–January 2022. Children aged < 18 years were enrolled if they were commencing or had already commenced antimicrobial therapy for lower respiratory tract infection. mini-BAL: non-bronchoscopic bronchoalveolar lavage

During the study period (April 2020–January 2022), 971 children were screened for study eligibility and 100 children were enrolled (Fig. 2).

### Patient characteristics

The most common primary diagnosis was respiratory illness (Table 1). This included bronchiolitis (46%), followed by pneumonia (25%), asthma/viral-induced wheeze (10%), and other respiratory illnesses (19%) including severe bronchopulmonary dysplasia, drowning, pulmonary oedema, structural airway problems, acute respiratory distress syndrome, and pneumonitis. Patients spent a mean 75% of days on the PICU (SD 27) receiving antimicrobial therapy.

**Table 1:** Demographic and clinical features of study participants

Variable	Total	Suspected CAP	Suspected HAP/VAP	*p*-value
	*N* = 100	*N* = 80	*N* = 20	
*Demographics*				
Age (years)—median (IQR)	1.2 (0.3–5.2)	1.1 (0.3–4.8)	2.8 (0.9–15.0)	0.072^a^
Sex (male)—*n* (%)	58	44 (55)	14 (70)	0.224^b^
Weight (kilograms)—median (IQR)	10.6 (5.3–19.7)	9.8 (5.0–18.5)	12.9 (9.0–41.2)	0.066^a^
Significant co-morbidity—*n* (%)	33	24 (30)	10 (50)	0.091^b^
PIM3 score—median (IQR)	3.2 (0.5–5.0)	2.0 (0.5–5.0)	3.6 (2.1–5.0)	0.099^a^
*Primary diagnostic category—n (%)*				
Respiratory	62	55 (69)	7 (35)	0.005^b^
Neurological	11	9 (11)	2 (10)	0.873^b^
Cardiovascular	7	5 (6)	2 (10)	0.346^b^
Trauma	6	4 (5)	2 (10)	0.709^b^
Post-operative care	6	1 (1)	5 (25)	< 0.001^b^
Other	8	6 (8)	2 (10)	0.712^b^
*Admission source—n (%)*				
Retrieval team	67	57 (71)	10 (50)	0.071^b^
Emergency department	16	14 (18)	2 (10)	0.670^b^
Paediatric ward	10	7 (9)	3 (15)	0.694^b^
Theatre and recovery	7	2 (3)	5 (25)	< 0.001^b^
*Clinical factors at time of sampling*				
Observations—mean (SD)				
Temperature (°C)	36.9 (1.1)	36.9 (1.2)	37.0 (0.7)	0.890^c^
Fraction of inspired oxygen (%)	44.1 (19.7)	43.2 (18.5)	47.6 (24.2)	0.450^c^
Mean airway pressure (cm H_2_O)	12.0 (3.3)	11.9 (3.2)	12.4 (3.8)	0.617^c^
Secretions—*n* (%)*				
Absent/minimal	29	27 (34)	2 (10)	0.034^b^
Present non-purulent	56	41 (52)	15 (75)	0.063^b^
Present purulent	14	11 (14)	3 (15)	0.902^b^
*Chest X-ray*—*n* (%)*				
Diffuse/patchy infiltrate	29	25 (32)	4 (20)	0.307^b^
Localised infiltrate	29	22 (28)	7 (35)	0.394^b^
No infiltrate	25	22 (28)	3 (15)	0.237^b^
Other	16	10 (13)	6 (30)	0.060^b^
*Haematology and biochemistry—median (IQR)*				
White cell count (10^9^/L)	8.5 (5.8–14.0)	8.6 (5.8–14.7)	8.0 (5.8–11.1)	0.520^a^
Neutrophils (10^9^/L)	4.7 (2.7–10.3)	5.0 (2.7–10.7)	4.3 (3.1–7.6)	0.597^a^
Lymphocytes (10^9^/L)	2.1 (1.0–3.2)	2.1 (1.0–3.2)	2.3 (1.1–3.3)	0.856^a^
Platelets (10^9^/L)	280 (183–413)	301 (217–423)	220 (112–259)	0.007^a^
C-reactive protein (mg/L)	28 (7–93)	26 (6–83)	58 (10–186)	0.210^a^
*Outcomes*				
Days free of treatment at 28 days–mean (SD)				
Antimicrobial therapy	16.8 (7.9)	21.1 (6.7)	13.6 (8.3)	0.002^c^
Mechanical ventilation	19.1 (7.2)	20.1 (6.5)	14.8 (8.6)	0.016^c^
Inotropes	26.1 (5.1)	26.2 (4.9)	25.6 (5.6)	0.640^c^
PICU admission	16.8 (7.9)	17.9 (7.6)	11.4 (7.6)	0.003^c^
Survival to hospital discharge—*n* (%)	94	77 (96)	17 (85)	0.058^b^

*CAP* community acquired pneumonia; *HAP* hospital acquired pneumonia; *IQR* interquartile range; *LRTI* lower respiratory tract infection; *PICU* paediatric intensive care unit; *SD* standard deviation; *VAP* ventilator-associated pneumonia

*Data missing for one patient in the medical record

^a^Mann–Whitney U test

^b^Chi-square test for independence

^c^Student’s *t*-test for equality of means, two-sided *p*, equal variance not assumed

### Bacterial and fungal detections on TAC

One hundred respiratory samples were obtained: 91 mini-BAL and 9 ETT aspirate samples (Additional file 1). 99/100 TAC passed quality control. Bacteria were detected more frequently on TAC (56/99 (57%)) than microbiology cultures (16/91(18%)) (*p* < 0.001) (Additional file 1: Table S2). This were also the case for fungi, found on 17/99 (17%) of TAC compared to 2/91 (2%) (*p* < 0.001) of microbiology cultures.

### Sensitivity and specificity of TAC

We excluded 9/100 (9%) of samples from the analysis in which microbiology culture was not obtained from the same sample as the TAC.

Positive microbiology cultures were reported in eighteen samples (Table S3). In four of these, there was a discrepancy with the TAC result. In one case, Coagulase-negative *Staphylococcus* was grown but not detected by TAC, although a linked detection of *mecA* (Ct 29) was noted. In two cases, TAC detected the organism but above our cut-off Ct of ≥ 32 (*Pseudomonas aeruginosa* with a Ct of 37 and an *Enterococcus faecalis* with a Ct of 35). The fourth was an off-panel organism, *Candida dubliniensis*; however, this was associated with genus-level Candida spp (Ct 23) and pan-fungal 18 s rRNA gene (Ct 24).

Using our predefined criteria, TAC sensitivity was 89.5% (CI_95_ 66.9–98.7), with a specificity of 97.9% (CI_95_ 97.2–98.5), a positive likelihood ratio of 43.4 (CI_95_ 31.2–60.5), and negative likelihood ratio of 0.1 (CI_95_ 0.0–0.4) (Additional file 1: Fig. S1).

### Time to result

**Fig. 3:**
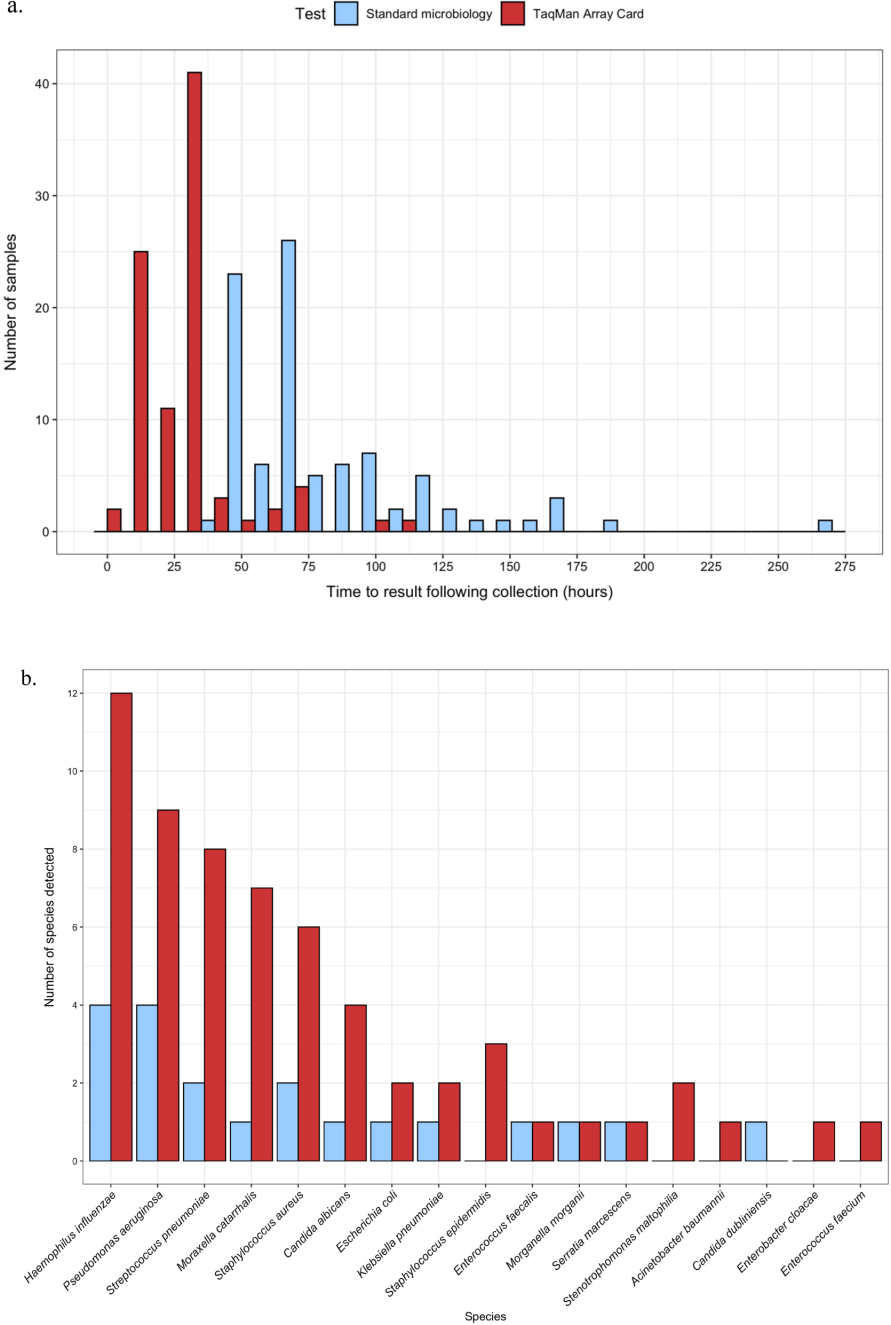
Performance of TaqMan Array Card (TAC) in the detection of bacterial and fungal micro-organisms compared to routine microbiology culture. **A** Time to reported result in hours from the time the sample was collected. **B** Number of detections of bacterial and fungal micro-organisms using TaqMan Array Card (TAC) with cycle threshold result ≤ 32 compared to standard microbiology culture

TAC took a median of 25.8 h (IQR 9.1–29.8 h) from sample collection to a reported result and 8.9 h (IQR 5.5–24.3 h) from laboratory registration to result (Fig. 3a). Cultures took a median 110.4 h (IQR 85.2–141.6 h) from sample collection to a reported positive result and 69.4 h (IQR 52.5–78.6 h) from sample collection to a reported negative result. Compared with microbiology culture, TAC was significantly quicker for both positive (Z = -3.593, *p* < 0.001) and negative results (*Z* = − 7.424, *p* < 0.001).

### Positive results reported by TAC only

An additional 44 bacterial and fungal species (in 29 samples) were detected on TAC at Ct ≤ 32 (median Ct 28.0, IQR 26.0–30.8) (Fig. 3b). They included *Haemophilus influenzae* (*n* = 8), *P. aeruginosa* (*n* = 6), *Moraxella catarrhalis* (*n* = 6), *Streptococcus pneumoniae* (*n* = 6), *Staphylococcus aureus* (*n* = 4), *Staphylococcus epidermidis* (*n* = 3), *Candida albicans* (*n* = 3), *Stenotrophomonas maltophilia* (*n* = 2), and individual samples with *Acinetobacter baumannii*, *E. faecalis*, *Enterococcus faecium*, *Enterobacter cloacae*, *Escherichia coli*, and *Klebsiella pneumoniae*.

TAC identified an additional 35 bacterial species, validated using 16S sequencing, that were not identified by microbiology culture (Additional file 1: Table S4), representing a 184% relative increase in detection. The Ct value of bacterial species was lower in those that had a corresponding positive culture (Ct 22.5, IQR 20.0–27.0) in comparison with those that were culture negative and confirmed with 16S sequencing (Ct 28.0, IQR 26.0–30.0, *p* < 0.001). Five bacterial species were identified by TAC that were not identified by culture nor met the 16S sequencing validation criteria (median Ct 28.0, IQR 27.5–31.5).

### Viral detections on TAC

Viral targets on the TAC were not formally validated against respiratory virus PCR due to reduced availability of the reference test during the COVID-19 pandemic. However, at least one viral pathogen was found on 46/99 (46%) TACs with Ct ≤ 32 (Additional file 1: Table S2). There were 31 patients who received a routine viral respiratory multiplex test, of which 22/31 (71%) were concordant with TAC results (Additional file 1: Table S5). Concordance increased to 6/7 (86%) restricting to ETT aspirate and mini-BAL samples, matching the sampling method used for TAC.

### The impact of TAC on antimicrobial decision-making

**Fig. 4:**
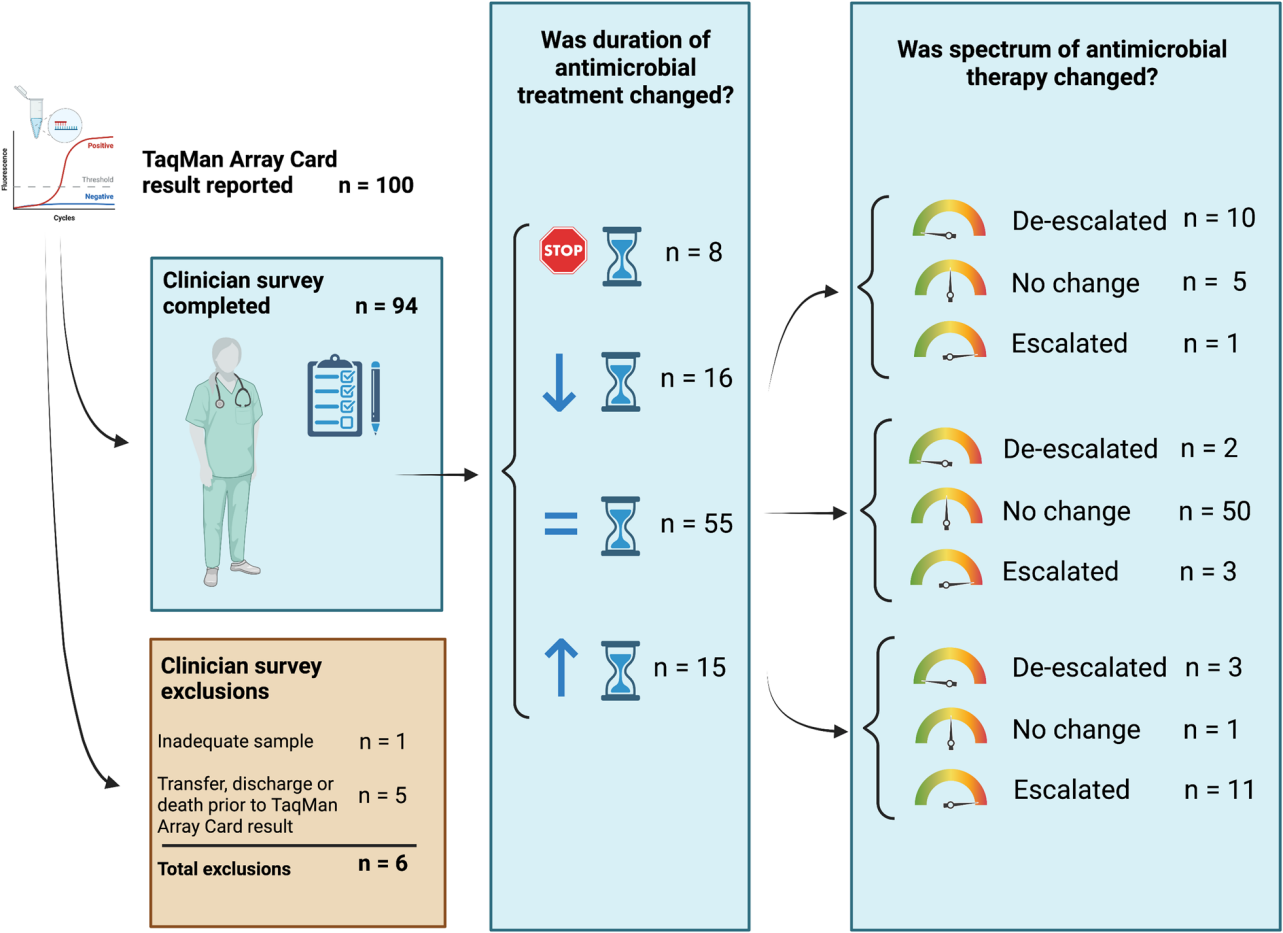
Actions of consultants with respect antimicrobial prescribing after reported TAC results. Critically unwell children underwent custom TaqMan Array Card (TAC) for suspected respiratory infection. Paediatric intensive care consultants were surveyed in respect to their prescribing once the results for TAC were documented in the electronic medical record. There were a range of changes made to antimicrobial prescriptions—in both the duration of treatment delivered and the spectrum of antimicrobial therapy

Consultants reported a change in prescription in 44/94 (47%) of cases based on reported TAC results. Antimicrobial therapy duration was reduced or stopped in 24/94 (26%) of children, and prolonged in 15/94 (16%) of children (Fig. 4). Of the 86 children that continued antimicrobial therapy, the spectrum of treatment was broadened in 15/86 (17%) and reduced in 15/86 (17%).

### The impact of TAC on total antimicrobial prescriptions

Due to a higher proportion of suspected VAP in our prospective cohort (Additional file 1: Table S6), the case–control analysis of antimicrobial utilisation was limited to children with suspected CAP (Additional file 1: Table S7). We found no significant difference in the proportion of days on antimicrobial therapy in children with suspected CAP in our cohort (*n* = 80, 75.9% mean PICU days, SD 33.0) compared to the controls (*n* = 49, 65.9% mean PICU days, SD 29.3). Additionally, we found no significant difference in ASI between the those in our cohort (median 38.5, IQR 23.3–58.9), versus controls (ASI 30.0, IQR 18.0–64.5, *Z* = − 1.084, *p* = 0.298). We did, however, observe a reduction in the use of aminoglycosides (2.6% vs. 5.1% treatment days) and macrolides (16.6% vs. 21.9% treatment days) in our cohort with suspected CAP compared to controls; this trend was reversed for beta-lactams (61.4% vs. 54.4% treatment days) (Additional file 1: Table S8).

## Discussion

In this study, we found that TAC identified bacteria and fungi more quickly than routine microbiology culture and increased the detection of bacterial species substantially. This result is important in view of the low yield of respiratory cultures in PICU, which may be compounded by the early administration of broad-spectrum antimicrobials and lack of routine culture techniques for atypical organisms. Having a diagnostic test with greater sensitivity for bacterial organisms has the potential to reassure clinicians that they can identify bacterial co-infection.

The present study determined a similar sensitivity and specificity of TAC as compared to adults with suspected VAP (sensitivity 92% (CI_95_ 83–98) and specificity 97% (CI_95_ 97–98)) [10]. This suggests that the smaller volume mini-BAL samples obtained in children are still reliable when compared to bronchoscopic samples obtained in adults. Of the four organisms found on culture, but were not significant on TAC, there were two that were likely commensals (*E. faecalis* and *C. dubliniensis*). We utilised 16S sequencing to assess the detection of additional bacteria by TAC, an approach that is only accurate to genus level of taxonomy. Of these additional bacteria identified, 35/40 (87.5%) were highly likely to be true positives according to the 16S sequencing data, whilst qPCR is more sensitive than sequencing, we cannot be certain about the validity of the remaining five (12.5%).

Numerous adult studies have found rapid molecular tests to be sensitive in detection of respiratory pathogens [21–25], but they have not yet been clinically evaluated in critically ill children. TAC was slower that other reported tests such as the Biofire Film Array (bioMérieux) and Unyvero Pneumonia Panel (Curetis) [23, 26]. However, our study was conducted in a clinical setting, with laboratory staff only available to process samples in business hours, which was a factor in the time from clinical sampling to result.

Consultants reported that they changed both the spectrum and the duration of antimicrobial therapy based on 47% of TAC results. This was not reflected in a reduction in the proportion of PICU days spent on antimicrobial therapy or the burden of treatment according to total ASI in children with CAP. There are several potential reasons for this. We measured total antimicrobial prescriptions, not just prescriptions for LRTI. Given that critically ill children are often treated for multi-system infections, the impact of the test may have been masked. Additional antimicrobial therapy may have been administered due to false positives or due to the high sensitivity of TAC result and the possibility that colonisation is reported as infection. Escalation of therapy is beneficial in the avoidance of antimicrobial resistance (AMR) if it prevents under treatment of infection. We therefore suggest that future studies evaluate the number of days in which patients receive appropriate antimicrobial therapy in addition to assessing total antimicrobial use.

Given that TAC is highly sensitive, mini-BAL was advised in the protocol to avoid irrelevant pathobionts found in the upper airway and ETT biofilms. Previous reports have predominantly used swabs or tracheal aspirates [8, 26, 27]. Nine samples were obtained via ETT aspiration despite this. In addition, samples were obtained by a range of physiotherapy, nursing, and medical staff. These factors may have introduced sample heterogeneity but did not impact on the validity of our findings given that analysis was carried out only on samples where TAC and microbiology culture were completed on the same specimen.

An alternative rapid diagnostic method is next-generation metagenomic sequencing (NGS), which has the advantage of being unrestricted in the potential pathogens it may detect. Paediatric studies of NGS describe it as a sensitive test, but it is difficult to determine its clinical application given that studies tend to report positive detection rates in comparison with culture rather than sensitivity and specificity [28–30]. The relevance of low prevalence pathogens and fluctuations in the composition of the respiratory microbiome remains a challenge for the interpretation of molecular diagnostics [31, 32].

It was not possible to reliably measure the performance of TAC for the detection of viral species in comparison with routine multiplex qPCR testing. This was firstly due to the PICU having severely restricted access to viral investigations during the COVID-19 pandemic, during which the laboratory prioritised SARS-CoV-2 testing. Secondly, viruses are shed more heavily in the upper airway than lower airway; hence, discordance between these sample locations is common [33]. Only 7/31 children enrolled to RASCALS that received routine viral multiplex tests had samples obtained from the lower airway; hence, caution should be applied to the comparison with samples which underwent TAC. Respiratory viral RNA can be shed for many weeks after the virus is viable; hence, implications of a positive test can be unclear [34]. Given that bacterial co-infection occurs in up to 33% of CAP [35] and 38% of bronchiolitis [36], the detection of viral species alone is unlikely to reassure PICU clinicians that antimicrobial therapy may be ceased [37]. Given that viral respiratory multiplex qPCR tests have been extensively investigated [38], this study focused on bacterial and fungal pathogen detection to aid antimicrobial decision-making.

This study was not large enough to analyse the Ct threshold at which growth occurred on microbiology culture for specific organisms; therefore, we reported the presence of any organism. A future study investigating what is found by TAC in respiratory samples from patients without clinical suspicion of pneumonia may help further determine significance of micro-organisms detected at low levels (i.e. high Ct values) which may represent colonisation rather than infection.

TAC is unique among commercially available rapid diagnostic tests [6, 26], in that the configuration of the card can be adapted according to the needs of the PICU. We modified the TAC to incorporate diagnostic primers for SARS-CoV-2 during the pandemic. Depending on the number of targets desired per sample, multiple samples can be loaded onto the card to improve cost-effectiveness. This flexibility allows the card to be used for a range of medical indications and be tailored to local pathogens. However, this is currently only possible in research studies as it is not an accredited in vitro diagnostic (IVD) assay.

To our knowledge, this is the first study of a rapid diagnostic test for bacterial and fungal causes of both CAP and VAP in ventilated, critically ill children. We propose TAC be used as an adjunct rather than a replacement for microbiology culture in services which have a microbiology facility that can assist with test interpretation. TAC may provide an early diagnosis to direct antimicrobial therapy, but microbiological cultures should occur in parallel to provide antimicrobial susceptibility data.

## Conclusions

TAC can be used to reliably detect pathogens quicker than routine culture in critically ill children with suspected LRTI. The equipment required to undertake TAC is likely to be available in most tertiary hospitals with a molecular diagnostic laboratory. A multi-centre study to determine actions following TAC report, including the impact of viral pathogen detection, would be beneficial for determining whether TAC should have more widespread implementation. Future studies should incorporate antimicrobial decision support and economic analysis.

## Supplementary Information

**Additional file 1**. Additional methods and results for the Rapid Assay for Sick Children with Acute Lung infection Study.

## Data Availability

With the exception of potentially identifying information, study data are available at the Open Science Framework: The rapid detection of respiratory pathogens in critically ill children [Internet]. OSF; 2022. Available from: osf.io/yczuf.

## References

[1] (2022) Paediatric Intensive Care Audit Network Annual Report (2021)

[2] NHS England (2021) National schedule of NHS costs—year 2020–2021—NHS trusts and NHS foundation trusts

[3] Pandolfo AM, Horne R, Jani Y (2021). Understanding decisions about antibiotic prescribing in ICU: an application of the necessity concerns framework. BMJ Qual Saf.

[4] Harris AM, Bramley AM, Jain S (2017). Influence of antibiotics on the detection of bacteria by culture-based and culture-independent diagnostic tests in patients hospitalized with community-acquired pneumonia. Open Forum Infect Dis.

[5] Clark J, White D, Daubney E (2021). Low diagnostic yield and time to diagnostic confirmation results in prolonged use of antimicrobials in critically ill children. Wellcome Open Res.

[6] Darie AM, Khanna N, Jahn K (2022). Fast multiplex bacterial PCR of bronchoalveolar lavage for antibiotic stewardship in hospitalised patients with pneumonia at risk of Gram-negative bacterial infection (Flagship II): a multicentre, randomised controlled trial. Lancet Respir Med.

[7] High J, Enne VI, Barber JA (2021). INHALE: the impact of using FilmArray Pneumonia Panel molecular diagnostics for hospital-acquired and ventilator-associated pneumonia on antimicrobial stewardship and patient outcomes in UK critical care-study protocol for a multicentre randomised controlled trial. Trials.

[8] Liu K, Jing H, Chen Y (2020). Evaluation of TaqMan Array card (TAC) for the detection of 28 respiratory pathogens. BMC Infect Dis.

[9] Kodani M, Yang G, Conklin LM (2011). Application of TaqMan low-density arrays for simultaneous detection of multiple respiratory pathogens. J Clin Microbiol.

[10] Navapurkar V, Scott JB, Maes M (2022). Development and implementation of a customised rapid syndromic diagnostic test for severe pneumonia [version 3; peer review: 2 approved]. Wellcome Open Res.

[11] Maes M, Higginson E, Pereira-Dias J (2021). Ventilator-associated pneumonia in critically ill patients with COVID-19. Crit Care.

[12] Clark JA, Kean IRL, Curran MD (2021). Rapid Assay for Sick Children with Acute Lung infection Study (RASCALS): diagnostic cohort study protocol. BMJ Open.

[13] Harris PA, Taylor R, Minor BL (2019). The REDCap consortium: Building an international community of software platform partners. J Biomed Inform.

[14] Straney L, Clements A, Parslow RC (2013). Paediatric index of mortality 3: an updated model for predicting mortality in pediatric intensive care*. Pediatr Crit Care Med.

[15] Yehya N, Harhay MO, Curley MAQ (2019). Reappraisal of ventilator-free days in critical care research. Am J Respir Crit Care Med.

[16] Public Health England (2019) UK standards for microbiology investigations: investigation of bronchoalveolar lavage, sputum and associated specimens.

[17] R Studio Team (2022) RStudio: integrated development for R.

[18] Wickham H (2016). ggplot2: elegant graphics for data analysis.

[19] Sullivan BA, Panda A, Wallman-Stokes A (2021). Antibiotic spectrum index: a new tool comparing antibiotic use in three NICUs. Infect Control Hosp Epidemiol.

[20] Gerber JS, Hersh AL, Kronman MP (2017). Development and application of an antibiotic spectrum index for benchmarking antibiotic selection patterns across hospitals. Infect Control Hosp Epidemiol.

[21] Murphy CN, Fowler R, Balada-Llasat JM (2020). Multicenter evaluation of the BioFire FilmArray pneumonia/pneumonia plus panel for detection and quantification of agents of lower respiratory tract infection. J Clin Microbiol.

[22] Buchan BW, Windham S, Balada-Llasat J-M (2020). Practical comparison of the BioFire FilmArray pneumonia panel to routine diagnostic methods and potential impact on antimicrobial stewardship in adult hospitalized patients with lower respiratory tract infections. J Clin Microbiol.

[23] Webber DM, Wallace MA, Burnham C-AD, Anderson NW (2020). Evaluation of the BioFire FilmArray pneumonia panel for detection of viral and bacterial pathogens in lower respiratory tract specimens in the setting of a tertiary care academic medical center. J Clin Microbiol.

[24] Lee SH, Ruan S-Y, Pan S-C (2019). Performance of a multiplex PCR pneumonia panel for the identification of respiratory pathogens and the main determinants of resistance from the lower respiratory tract specimens of adult patients in intensive care units. J Microbiol Immunol Infect.

[25] Timbrook TT, Hueth KD, Ginocchio CC (2021). Identification of bacterial co-detections in COVID-19 critically Ill patients by BioFire^®^ FilmArray^®^ pneumonia panel: a systematic review and meta-analysis. Diagn Microbiol Infect Dis.

[26] Enne VI, Aydin A, Baldan R (2022). Multicentre evaluation of two multiplex PCR platforms for the rapid microbiological investigation of nosocomial pneumonia in UK ICUs: the INHALE WP1 study. Thorax.

[27] Weinberg GA, Schnabel KC, Erdman DD (2013). Field evaluation of TaqMan Array Card (TAC) for the simultaneous detection of multiple respiratory viruses in children with acute respiratory infection. J Clin Virol.

[28] Guo W, Cui X, Wang Q (2022). Clinical evaluation of metagenomic next-generation sequencing for detecting pathogens in bronchoalveolar lavage fluid collected from children with community-acquired pneumonia. Front Med.

[29] Yang A, Chen C, Hu Y (2022). Application of metagenomic next-generation sequencing (mNGS) using bronchoalveolar lavage fluid (BALF) in diagnosing pneumonia of children. Microbiol Spectr.

[30] Wang H, Lu Z, Bao Y (2020). Clinical diagnostic application of metagenomic next-generation sequencing in children with severe nonresponding pneumonia. PLoS ONE.

[31] Charalampous T, Kay GL, Richardson H (2019). Nanopore metagenomics enables rapid clinical diagnosis of bacterial lower respiratory infection. Nat Biotechnol.

[32] Zheng Y, Qiu X, Wang T, Zhang J (2021). The diagnostic value of metagenomic next-generation sequencing in lower respiratory tract infection. Front Cell Infect Microbiol.

[33] Boonyaratanakornkit J, Vivek M, Xie H (2019). Predictive value of respiratory viral detection in the upper respiratory tract for infection of the lower respiratory tract with hematopoietic stem cell transplantation. J Infect Dis.

[34] Cevik M, Tate M, Lloyd O (2021). SARS-CoV-2, SARS-CoV, and MERS-CoV viral load dynamics, duration of viral shedding, and infectiousness: a systematic review and meta-analysis. Lancet Microbe.

[35] Cevey-Macherel M, Galetto-Lacour A, Gervaix A (2009). Etiology of community-acquired pneumonia in hospitalized children based on WHO clinical guidelines. Eur J Pediatr.

[36] Wiegers HMG, van Nijen L, van Woensel JBM (2019). Bacterial co-infection of the respiratory tract in ventilated children with bronchiolitis; a retrospective cohort study. BMC Infect Dis.

[37] Pandolfo AM, Horne R, Jani Y (2021). Intensivists’ beliefs about rapid multiplex molecular diagnostic testing and its potential role in improving prescribing decisions and antimicrobial stewardship: a qualitative study. Antimicrob Resist Infect Control.

[38] Huang H-S, Tsai C-L, Chang J (2018). Multiplex PCR system for the rapid diagnosis of respiratory virus infection: systematic review and meta-analysis. Clin Microbiol Infect.

