# Supplementary material for: The rapid detection of respiratory pathogens in critically ill children
Source: Crit Care. 2023 Jan 10;27:11. doi: 10.1186/s13054-023-04303-1 (PMC9831374; doi:10.1186/s13054-023-04303-1)
Supplement: Supplementary file 1 — Additional file 1. Additional methods and results for the Rapid Assay for Sick Children with Acute Lung infection Study. [file 13054_2023_4303_MOESM1_ESM.docx]

Supplementary materials: The rapid detection of respiratory pathogens in critically ill children.

John A Clark MBBS MRCPCH ^1, 2*^, Andrew Conway Morris PhD ^2,3,4^, Martin D Curran PhD ^5^, Deborah White MSc ^2^, Esther Daubney BSc ^2^, Iain RL Kean PhD ^1^, Vilas Navapurkar FFICM ^2^, Josefin Bartholdson Scott PhD ^6^, Mailis Maes PhD ^6^, Rachel Bousfield BMBS ^2, 5^, M. Estée Török PhD FRCP FRCPath ^2, 7^, David Inwald PhD FRCPCH ^2^, Zhenguang Zhang PhD ^1^, Shruti Agrawal MD FRCPCH ^1,2^, Constantinos Kanaris PhD FRCPCH FFICM ^2, 8^, Fahad Khokhar BSc ^6^, Theodore Gouliouris PhD FRCPath ^2^_,_ ^5^, Stephen Baker PhD ^6^, and Nazima Pathan PhD FRCPCH ^1, 2*^

1. Department of Paediatrics, University of Cambridge
2. Cambridge University Hospitals NHS Foundation Trust
3. Division of Anaesthesia, Department of Medicine, University of Cambridge
4. Division of Immunology, Department of Pathology, University of Cambridge
5. United Kingdom Health Security Agency, Clinical Microbiology and Public Health Laboratory, Cambridge
6. Cambridge Institute of Therapeutic Immunology and Infectious Disease, University of Cambridge
7. Division of Infectious Diseases, Department of Medicine, University of Cambridge
8. Blizard Institute, Queen Mary University of London

*Corresponding author: Dr John Clark, Department of Paediatrics, Level 8, Addenbrooke’s Hospital, Cambridge Biomedical Campus, Cambridge CB2 0QQ. Email: [jac302@cam.ac.uk](mailto:jac302@cam.ac.uk)

**Sequencing methods**

Library preparation and sequencing was undertaken by NovoGene Bioinformatics Technology Co. Ltd. Pre-processing of data included merging paired-end reads using FLASH (v1.2.7) and filtering of raw tags using Qiime (v1.7.0) [1, 2]. Taxonomy was reported according to the reference SILVA138 database using UCHIME algorithm and chimera sequences were removed [2, 3]. Sequence analysis was performed using Uparse (v7.0.1090), with **≥**97% similarity assigned to the same Operational Taxonomic Units (OTUs) [4]. Species annotation was undertaken using Mothur method against the SSUrRNA database of SILVA138 [5–7]. OTUs belonging to known environmental contaminants were excluded from the analysis [8] To ensure organisms reported for comparison to TAC were highly representative of the sample, results were filtered to genus which were present in **≥**500 reads and represented **≥**0.5% of the classified reads in the sample. To validate detections on TAC that were not identified on culture, all targets on TAC at either species or genus level were included.

**Table S1** Additional antimicrobials added to Antimicrobial Spectrum Index

| Drug | MSSA | Enterococcus spp. | Anaerobic organisms | Bacteroides fragilis | Moraxella spp./*Haemophilus* *influenzae* | Escherichia coli/Klebsiella spp. | Enterobacter/Serratia/Citrobacter | Extended spectrum beta lactamase producing organisms | Pseudomonas | MRSA | Penicillin-resistant streptococcus | Atypical respiratory pathogens^$^ | MDRO | **Total** |
| --- | --- | --- | --- | --- | --- | --- | --- | --- | --- | --- | --- | --- | --- | --- |
| Ceftolozane/tazobactam | 0 | 0 | 1 | 1 | 1 | 1 | 1 | 1 | 1 | 0 | 0 | 0 | 1 | 8 |
| Flucloxacillin | 1 | 0 | 0 | 0 | 0 | 0 | 0 | 0 | 0 | 0 | 0 | 0 | 0 | 1 |
| Teicoplanin | 1 | 1 | 0 | 0 | 0 | 0 | 0 | 0 | 0 | 1 | 0 | 0 | 0 | 3 |

^MDRO: Multi-drug resistant organism; MRSA: Methicillin resistant staphylococcus aureus; MSSA: Methicillin sensitive staphylococcus aureus. $^ *^Chlamydia pneumoniae, mycoplasma pneumoniae^* ^and^ *^Legionella pneumophila^* ^NB: This table is an addition to the original Antimicrobial Spectrum Index developed by Gerber et al 2017 [9].^

**Additional results**

*Mini-BAL samples*

Due to the precautions associated with aerosol generating procedures during the COVID-19 pandemic, sample volumes could only be measured after aliquots were taken for routine investigations and TAC, which was possible in 84/91 (92%) of mini-BAL samples. There was a mean of 2,294 µL (SD 1,156) sample remaining after microbiology culture, which exceeded the 750 µL volume required for nucleic acid extraction. No adverse events were reported relating to sample collection. There was significant microbiological growth in 18/91(20%) of samples which underwent both microbiology culture and TAC. Microbiology culture was more likely to be positive in patients with suspected VAP (8/17) than CAP (10/74), (Χ^2^ 9.8037, *p*=0.002).

*TAC*

TAC passed quality control in 99/100 assays. One assay failed as no human DNA was detected in the sample and no growth was detected on culture. This sample was excluded from sensitivity and specificity analysis. Excluding controls there were a total 496/9,108 (5.4%) hits on TAC with a mean Ct value of 29.5 (SD 5.2). Of these, 253 (63%) had a Ct value of ≤32. There was at least one detection with Ct ≤32 in 78/99 (79%) samples. Based on species specific targets, these results represented a median of one (IQR 0-2) bacterial species and one (IQR 0 -1) viral species detected per patient.

**Table S2** Detections on TaqMan Array Card compared to routine investigations.

| **Organism** | **Taxonomic rank** | **Name** | **Detected on TAC (n/total)** | **Detected on microbiology culture/virology (n/total)** | **p-value^#^** |
| --- | --- | --- | --- | --- | --- |
| Bacteria | Kingdom/  genus/  family | 16S rRNA gene^±^ | 2/38 | * | N/A |
|  |  | Coagulase negative *Staphylococcus* | 8/99 | 1/91 | 0.036 |
|  |  | *Enterobacteriaceae* | 10/99 | * | N/A |
|  |  | *Legionella* spp | 0/61 | * | N/A |
|  |  | *Staphylococcus* PVL | 0/99 | * | N/A |
|  |  | *Streptococcus* spp | 33/99 | * | N/A |
|  | AMR gene | *MecA* gene | 7/99 | * | N/A |
|  | Species | *Acinetobacter baumannii* | 1/99 | 0/91 | 1 |
|  |  | *Bacteroides fragilis* | 0/99 | 0/91 | 1 |
|  |  | *Bordetella pertussis* | 0/99 | 0/91 | 1 |
|  |  | *Chlamydia pneumoniae* | 0/99 | 0/91 | 1 |
|  |  | *Chlamydia psittaci* | 1/99 | 0/91 | 1 |
|  |  | *Coxiella burnetii* | 0/99 | 0/91 | 1 |
|  |  | *Enterobacter cloacae* | 1/99 | 0/91 | 1 |
|  |  | *Enterobacteriacae proteus* | 1/99 | 0/91 | 1 |
|  |  | *Elizabethkingia meningoseptica* | 0/99 | 0/91 | 1 |
|  |  | *Enterococcus faecalis*^±^ | 1/38 | 1/91 | 0.504 |
|  |  | *Enterococcus faecium*^±^ | 1/38 | 1/91 | 0.504 |
|  |  | *Escherichia coli* | 2/99 | 1/91 | 1 |
|  |  | *Haemophilus influenzae* | 14/99 | 4/91 | 0.026 |
|  |  | *Klebsiella pneumoniae* | 3/99 | 1/91 | 0.062 |
|  |  | *Legionella pneumophilia* | 0/61 | 0/91 | 1 |
|  |  | *Leptospira* | 0/61 | 0/91 |  |
|  |  | *Moraxella catarrhalis* | 8/99 | 1/91 | 0.036 |
|  |  | *Morganella morganii* | 1/99 | 1/91 | 1 |
|  |  | *Mycoplasma pneumoniae* | 0/99 | 0/91 | 1 |
|  |  | *Mycobacterium tuberculosis* | 0/99 | 0/91 | 1 |
|  |  | *Neisseria meningitidis* | 0/99 | 0/91 | 1 |
|  |  | *Pseudomonas aeruginosa* | 11/99 | 4/91 | 0.109 |
|  |  | *Serratia marcescens* | 2/99 | 1/91 | 1 |
|  |  | *Staphylococcus aureus* | 6/99 | 2/91 | 0.282 |
|  |  | *Staphylococcus epidermidis* | 4/99 | 0/91 | 0.122 |
|  |  | *Streptococcus pneumoniae* | 8/99 | 2/91 | 0.103 |
|  |  | *Streptococcus pyogenes* | 0/99 | 0/91 | 1 |
|  |  | *Stenotrophomonas maltophilia* | 2/99 | 0/91 | 0.498 |
|  | **TOTAL** | **Any bacterial detection** | **56/99** | **16/91** | **<0.001** |
| Fungi | Kingdom/  genus | *Candida* spp | 11/99 | * | N/A |
|  |  | 18S rRNA gene | 15/99 | * | N/A |
|  |  | *Aspergillus* 28S gene | 0/99 | * | N/A |
|  | Species | *Aspergillus fumigatus* | 0/99 | 0/91 | 1 |
|  |  | *Candida albicans* | 4/99 | 1/91 | 0.371 |
|  |  | *Pneumocystis jirovecii* | 1/99 | 0/91 | 1 |
|  | **TOTAL** | **Any fungal detection** | **17/99** | **2/91** | **<0.001** |
| Virus | Species | *Adenovirus* | 0/99 | 2/31 | 0.055 |
|  |  | *Bocavirus* | 5/99 | $ | N/A |
|  |  | *Cytomegalovirus* | 1/99 | $ | N/A |
|  |  | *Enterovirus* | 2/99 | 2/31 | 0.241 |
|  |  | *Epstein barr virus* | 2/99 | 1/1 | 0.056 |
|  |  | *Herpes simplex virus* | 0/99 | 0/7^£^ | 1 |
|  |  | *Human coronavirus NL63* | 0/99 | 0/7^£^ | 1 |
|  |  | *Human coronavirus OC43* | 0/99 | 0/7^£^ | 1 |
|  |  | *Human coronavirus OOC43/HKU* | 0/99 | 0/7^£^ | 1 |
|  |  | *Human coronavirus 229E* | 0/99 | 0/7^£^ | 1 |
|  |  | *Human metapneumovirus* | 0/99 | 0/31 | 1 |
|  |  | *Human parainfluenza virus 1* | 0/99 | 0/31 | 1 |
|  |  | *Human parainfluenza virus 2* | 0/99 | 0/31 | 1 |
|  |  | *Human parainfluenza virus 3* | 1/99 | 1/31 | 0.422 |
|  |  | *Human parainfluenza virus 4* | 0/99 | 1/31 | 0.239 |
|  |  | *Influenza A (any subtype)* | 1/99 | 0/31 | 1 |
|  |  | *Influenza B* | 0/99 | 0/31 | 1 |
|  |  | *Parechovirus* | 0/99 | $ | N/A |
|  |  | *Rhinovirus* | 17/99 | 10/31 | 0.081 |
|  |  | *Respiratory syncytial virus (any subtype)* | 17/99 | 3/31 | 0.402 |
|  |  | *SARS-CoV-2* | 3/61 | 4/100 | 1 |
|  | **TOTAL** | **Any viral detection** | **46/99** | **27/100** | **<0.001** |
| ^TAC = TaqMan Array card; ±In enrolments occurring from 5 February 2021 onwards, these targets were replaced to include three targets for^ *^SARS-CoV-2^*^, one target for^ *^Leptospira^* ^and an additional target for^ *^Legionella pneumophilia^*^; # = Calculated with the Fisher’s exact test; * These targets are not reported on culture, as this would normally be at species level identification. $ These targets are not included in routine respiratory viral multiplex panels in this institution. £ Routine testing for these viruses became available part-way through the study with the introduction of the Biofire Respiratory 2.1 assay (BioMérieux) to the molecular laboratory. This table compares detection of microorganisms on custom TaqMan Array Card at a cycle threshold cut-off^ **^≤^**^32. Note that whilst 91 samples that underwent microbiology testing were undertaken on the same sample as TAC, the 31 samples that underwent routine viral multiplex testing were obtained from a range of sources (refer to table S5 for more details). The total number of species identified for bacteria, fungi and viruses do not match the total detection, this is due to some samples having multiple species detected, and some species identified on routine investigations that were not targets on TAC.^  **Table S3** Growth on microbiology culture versus detection on TaqMan Array Card   \| **Study ID** \| **Sample method** \| **Pathogen name** \| **TaqMan array confirmatory result and Ct value** \| **Species identified on TAC (Ct ≤32)** \| **Detection on TAC including non-specific targets (any Ct)** \| \| --- \| --- \| --- \| --- \| --- \| --- \| \| C001 \| Mini-BAL \| Coagulase-negative Staphylococcus (CoNS) \| *mecA*: 29 \| *🗴* \| ✓ \| \| C002 \| ETT aspirate \| *Klebsiella pneumoniae* \| *K. pneumoniae* #1: 28  *K. pneumoniae* #2: 28 \| ✓ \| ✓ \| \| C004 \| ETT aspirate \| *Candida albicans* \| *C. albicans*: 27  *Candida* spp: 25 \| ✓ \| ✓ \| \| C008 \| Mini-BAL \| *Pseudomonas aeruginosa* \| *P. aeruginosa*: 37 \| *🗴* \| ✓ \| \| C029 \| Mini-BAL \| *Enterococcus faecalis* \| *E. faecalis*: 35 \| *🗴* \| ✓ \| \| C032 \| Mini-BAL \| *Pseudomonas aeruginosa* \| *P. aeruginosa* #1: 27  *P. aeruginosa* #2: 27 \| ✓ \| ✓ \| \| C036 \| Mini-BAL \| *Moraxella catarrhalis* \| *M. catarrhalis*: 21 \| ✓ \| ✓ \| \| C037 \| Mini-BAL \| *Candida dubliniensis* \| *Candida* spp: 23  18S rRNA: 24 \| *🗴* \| ✓ \| \| C046 \| Mini-BAL \| *Staphylococcus aureus* \| *S. aureus* #1: 28  *S. aureus* #2: 29  CoNS: 32 \| ✓ \| ✓ \| \| C048 \| ETT aspirate \| *Morganella morganii* \| *M. morganii*: 25 \| ✓ \| ✓ \| \| C052 \| Mini-BAL \| *Pseudomonas aeruginosa* \| *P. aeruginosa*: 22  *P. aeruginosa*: 20 \| ✓ \| ✓ \| \| C057 \| Mini-BAL \| *Escherichia coli* \| *E. coli* #1: 30  *E. coli* #2: 28 \| ✓ \| ✓ \| \| C059 \| Mini-BAL \| *Staphylococcus aureus* \| *S. aureus* #1: 26  *S. aureus* #2: 27  CoNS: 28 \| ✓ \| ✓ \| \| *Haemophilus influenzae* \| *H. influenzae* #1: 19  *H. influenzae* #2: 19 \| ✓ \| ✓ \| \| C067 \| Mini-BAL \| *Serratia marcescens* \| *S. marcescens* #1: 23  *S. marcescens* #2: 21 \| ✓ \| ✓ \| \| C089 \| Mini-BAL \| *Haemophilus influenzae* \| *H. influenzae* #1: 18  *H. influenzae* #2: 19 \| ✓ \| ✓ \| \| *Streptococcus pneumoniae* \| *Streptococcus* spp #1: 27  *Streptococcus* spp #2: 25  *S. pneumoniae* #1: 27  *S. pneumoniae* #2: 26 \| ✓ \| ✓ \| \| C107 \| Mini-BAL \| *Haemophilus influenzae* \| *H. influenzae* #1: 19  *H. influenzae* #2: 20 \| ✓ \| ✓ \| \| C111 \| Mini-BAL \| *Streptococcus pneumoniae* \| *S. pneumoniae* #1: 24  *S. pneumoniae* #2: 23  *Streptococcus* spp #1: 26  *Streptococcus* spp #2: 23 \| ✓ \| ✓ \| \| *Haemophilus influenzae* \| *H. influenzae* #1: 20  *H. influenzae* #2: 21 \| ✓ \| ✓ \| \| C121 \| Mini-BAL \| *Pseudomonas aeruginosa* \| *P. aeruginosa* #1: 20  *P. aeruginosa* #2: 20 \| ✓ \| ✓ \| \| ^CoNS: Coagulase negative staphylococcus; Ct: Cycle threshold; ETT: Endotracheal tube; Mini-BAL: Non-bronchoscopic bronchoalveolar lavage; TAC: TaqMan Array Card^ \| \| \| \| \| \| | | | | | |

|  |  | Culture | |  |  |  | Culture | |  |  |  | Culture | |  |  |  | Culture | |  |
| --- | --- | --- | --- | --- | --- | --- | --- | --- | --- | --- | --- | --- | --- | --- | --- | --- | --- | --- | --- |
|  | ***A. baumannii*** | | | |  | ***B. fragillis*** | | | |  | ***B. pertussis*** | | | |  | ***E. proteus*** | | | |
|  |  | + | - |  |  |  | + | - |  |  |  | + | - |  |  |  | + | - |  |
| TAC | + | 0 | 1 | 1 |  | + | 0 | 0 | 0 |  | + | 0 | 0 | 0 |  | + | 0 | 0 | 0 |
|  | - | 0 | 90 | 90 |  | - | 0 | 91 | 91 |  | - | 0 | 91 | 91 |  | - | 0 | 91 | 91 |
|  |  | 0 | 91 |  |  |  | 0 | 91 |  |  |  | 0 | 91 |  |  |  | 0 | 91 |  |
|  |  |  |  |  |  |  |  |  |  |  |  |  |  |  |  |  |  |  |  |
|  | ***E. cloacae*** | | | |  | ***E. faecalis**** | | | |  | ***E. faecium**** | | | |  | ***E. meningoseptica*** | | | |
|  |  | + | - |  |  |  | + | - |  |  |  | + | - |  |  |  | + | - |  |
| TAC | + | 0 | 1 | 1 |  | + | 0 | 1 | 1 |  | + | 0 | 1 | 1 |  | + | 0 | 0 | 0 |
|  | - | 0 | 90 | 90 |  | - | 1 | 29 | 30 |  | - | 0 | 30 | 30 |  | - | 0 | 91 | 91 |
|  |  | 0 | 91 |  |  |  | 1 | 30 |  |  |  | 0 | 31 |  |  |  | 0 | 91 |  |
|  |  |  |  |  |  |  |  |  |  |  |  |  |  |  |  |  |  |  |  |
|  | ***E. coli*** | | | |  | ***H. influenzae*** | | | |  | ***K. pneumoniae*** | | | |  | ***L. pneumophila*** | | | |
|  |  | + | - |  |  |  | + | - |  |  |  | + | - |  |  |  | + | - |  |
| TAC | + | 1 | 1 | 2 |  | + | 4 | 8 | 12 |  | + | 1 | 1 | 2 |  | + | 0 | 0 | 0 |
|  | - | 0 | 89 | 89 |  | - | 0 | 79 | 79 |  | - | 0 | 89 | 89 |  | - | 0 | 91 | 91 |
|  |  | 1 | 90 |  |  |  | 4 | 87 |  |  |  | 1 | 90 |  |  |  | 0 | 91 |  |
|  |  |  |  |  |  |  |  |  |  |  |  |  |  |  |  |  |  |  |  |
|  | ***M. catarrhalis*** | | | |  | ***M. morganii*** | | | |  | ***M. tuberculosis*** | | | |  | ***N. meningitidis*** | | | |
|  |  | + | - |  |  |  | + | - |  |  |  | + | - |  |  |  | + | - |  |
| TAC | + | 1 | 6 | 7 |  | + | 1 | 0 | 1 |  | + | 0 | 0 | 0 |  | + | 0 | 0 | 0 |
|  | - | 0 | 84 | 84 |  | - | 0 | 90 | 90 |  | - | 0 | 91 | 91 |  | - | 0 | 91 | 91 |
|  |  | 1 | 90 |  |  |  | 1 | 90 |  |  |  | 0 | 91 |  |  |  | 0 | 91 |  |
|  |  |  |  |  |  |  |  |  |  |  |  |  |  |  |  |  |  |  |  |
|  | ***P. aeruginosa*** | | | |  | ***S. marcescens*** | | | |  | ***S. aureus*** | | | |  | ***S. epidermidis*** | | | |
|  |  | + | - |  |  |  | + | - |  |  |  | + | - |  |  |  | + | - |  |
| TAC | + | 3 | 6 | 9 |  | + | 1 | 0 | 1 |  | + | 2 | 4 | 6 |  | + | 0 | 3 | 3 |
|  | - | 1 | 81 | 82 |  | - | 0 | 90 | 90 |  | - | 0 | 85 | 85 |  | - | 0 | 88 | 88 |
|  |  | 4 | 87 |  |  |  | 1 | 90 |  |  |  | 2 | 89 |  |  |  | 0 | 91 |  |
|  |  |  |  |  |  |  |  |  |  |  |  |  |  |  |  |  |  |  |  |
|  | ***S. maltophilia*** | | | |  | ***S. pneumoniae*** | | | |  | ***S. pyogenes*** | | | |  | **Bacterial total** | | | |
|  |  | + | - |  |  |  | + | - |  |  |  | + | - |  |  |  | + | - |  |
| TAC | + | 0 | 2 | 2 |  | + | 2 | 6 | 8 |  | + | 0 | 0 | 0 |  | + | 16 | 41 | 57 |
|  | - | 0 | 89 | 89 |  | - | 0 | 83 | 83 |  | - | 0 | 91 | 91 |  | - | 2 | 1914 | 1916 |
|  |  | 0 | 91 |  |  |  | 2 | 89 |  |  |  | 0 | 91 |  |  |  | 18 | 1955 |  |
|  |  |  |  |  |  |  |  |  |  |  |  |  |  |  |  |  |  |  |  |
|  | ***C. albicans*** | | | |  | ***A. fumigatus*** | | | |  | **Fungal total** | | | |  | **Bacterial + Fungal total** | | | |
|  |  | + | - |  |  |  | + | - |  |  |  | + | - |  |  |  | + | - |  |
| TAC | + | 1 | 3 | 4 |  | + | 0 | 0 | 0 |  | + | 1 | 3 | 4 |  | + | 17 | 44 | 61 |
|  | - | 0 | 87 | 87 |  | - | 0 | 91 | 91 |  | - | 0 | 178 | 178 |  | - | 2 | 2092 | 2094 |
|  |  | 1 | 90 |  |  |  | 0 | 91 |  |  |  | 1 | 181 |  |  |  | 19 | 2136 |  |

**Fig. S1** Calculation data of custom TaqMan Array Card sensitivity and specificity for bacterial and fungal species.

^This figure demonstrates the individual and combined data used to determine the sensitivity and specificity of a custom TaqMan Array Card (TAC). Species that are not grown on standard microbiology culture have been excluded. These data are limited to bacterial and fungal species on the card, where cycle threshold result was ≤32, and excludes higher taxonomic targets and viruses. Positive and negative results on TAC are compared to microbiology culture. * These species were replaced on the TAC for SARS-CoV-2 targets during the study.^

**Table S4** Validation of bacterial species identified on TaqMan Array Card using 16S rRNA gene sequencing

| **Study ID** | **Detection on TaqMan Array Card** | **Cycle threshold(s)** | **Detection on culture** | **Validation by 16S rRNA gene sequencing*** | **Corresponding 16S rRNA gene sequencing reads**  **(n, % total classified reads)** | |  |
| --- | --- | --- | --- | --- | --- | --- | --- |
| C001 | *E. faecium* | *27/27* | *🗴* | *🗴* | *60* | (0.04) |  |
| C002 | *K. pneumoniae* | *28/29* | *🗸* | *🗴* | *0* | (0.00) |  |
| C021 | *S. pneumoniae* | *31/29* | *🗴* | *🗸* | *2242* | (2.42) |  |
|  | *H. influenzae* | *27/28* | *🗴* | *🗸* | *866* | (0.93) |  |
|  | *M. catarrhalis* | *26* | *🗴* | *🗸* | *2869* | (3.09) |  |
| C025 | *A. baumannii* | *23/19* | *🗴* | *🗸* | *63714* | (67.87) |  |
|  | *E. faecalis* | *29* | *🗴* | *🗸* | *621* | (0.66) |  |
|  | *S. maltophilia* | *28* | *🗴* | *🗴* | *4* | (0.00) |  |
| C032 | *P. aeruginosa* | *27/27* | *🗸* | *🗸* | *17737* | (26.71) |  |
| C035 | *M. catarrhalis* | *29* | *🗴* | *🗸* | *22921* | (40.58) |  |
| C036 | *M. catarrhalis* | *21* | *🗸* | *🗸* | *63354* | (87.07) |  |
| C037 | *P. aeruginosa* | *23/20* | *🗴* | *🗸* | *45361* | (57.41) |  |
| C042 | *S. epidermidis* | *28* | *🗴* | *🗸* | *4279* | (9.08) |  |
|  | *S. maltophilia* | *28* | *🗴* | *🗸* | *2975* | (6.31) |  |
| C046 | *S. pneumoniae* | *26/26* | *🗴* | *🗸* | *17625* | (21.29) |  |
|  | *H. influenzae* | *24/24* | *🗴* | *🗸* | *45805* | (55.33) |  |
|  | *S. aureus* | *28/29* | *🗸* | *🗸* | *1008* | (1.22) |  |
| C048 | *E. coli* | *31* | *🗴* | *🗸* | *1012* | (1.09) |  |
|  | *P. aeruginosa* | *31* | *🗴* | *🗸* | *1462* | (1.57) |  |
|  | *E. cloacae* | *28/32* | *🗴* | *🗴* | *197* | (0.21) |  |
|  | *M. morganii* | *25* | *🗸* | *🗸* | *27358* | (29.43) |  |
| C049 | *P. aeruginosa* | *31* | *🗴* | *🗸* | *4848* | (10.18) |  |
| C052 | *P. aeruginosa* | *22/20* | *🗸* | *🗸* | *92889* | (90.47) |  |
| C057 | *E. coli* | *30/28* | *🗸* | *🗸* | *18574* | (34.03) |  |
| C059 | *S. epidermidis* | *28* | *🗴* | *🗸* | *579* | (0.66) |  |
|  | *S. aureus* | *26/27* | *🗸* | *🗸* | *579* | (0.66) |  |
|  | *H. influenzae* | *19/19* | *🗸* | *🗸* | *63302* | (72.05) |  |
| C062 | *S. epidermidis* | *31* | *🗴* | N/A | N/A | |  |
| C063 | *P. aeruginosa* | *27/26* | *🗴* | *🗸* | *46063* | (58.18) |  |
|  | *M. catarrhalis* | *23* | *🗴* | *🗸* | *8366* | (10.57) |  |
| C067 | *K. pneumoniae* | *28/29* | *🗴* | *🗴* | *0* | (0.00) |  |
|  | *S. marcescens* | *23/21* | *🗸* | *🗸* | *41176* | (65.26) |  |
| C074 | *S. aureus* | *29/30* | *🗴* | *🗸* | *1064* | (4.68) |  |
| C078 | *M. catarrhalis* | *28* | *🗴* | *🗸* | *11538* | (21.32) |  |
| C084 | *H. influenzae* | *26/27* | *🗴* | *🗸* | *747* | (1.25) |  |
| C089 | *H. influenzae* | *18/19* | *🗸* | *🗸* | *3203* | (5.36) |  |
|  | *S. pneumoniae* | *27/26* | *🗸* | *🗸* | *69857* | (76.60) |  |
| C093 | *S. pneumoniae* | *31* | *🗴* | *🗸* | *31307* | (37.35) |  |
|  | *H. influenzae* | *24/26* | *🗴* | *🗸* | *29454* | (35.14) |  |
| C099 | *H. influenzae* | *27/29* | *🗴* | *🗸* | *35855* | (54.46) |  |
| C100 | *S. pneumoniae* | *29/32* | *🗴* | *🗸* | *3609* | (3.61) |  |
| C101 | *S. pneumoniae* | *31/31* | *🗴* | *🗸* | *13653* | (15.60) |  |
| C102 | *S. pneumoniae* | *31* | *🗴* | *🗸* | *1321* | (2.73) |  |
| C103 | *H. influenzae* | *31/29* | *🗴* | *🗸* | *3809* | (10.57) |  |
|  | *M. catarrhalis* | *27* | *🗴* | *🗸* | *582* | (1.62) |  |
| C107 | *H. influenzae* | *19/20* | *🗸* | *🗸* | *69776* | (77.84) |  |
| C109 | *M. catarrhalis* | *20* | *🗴* | *🗸* | *61502* | (74.41) |  |
| C111 | *S. pneumoniae* | *24/23* | *🗸* | *🗸* | *8230* | (8.64) |  |
|  | *H. influenzae* | *20/21* | *🗸* | *🗸* | *71303* | (74.83) |  |
| C114 | *H. influenzae* | *28/29* | *🗴* | *🗸* | *41880* | (74.73) |  |
| C115 | *S. aureus* | *29/30* | *🗴* | *🗸* | *10897* | (17.07) |  |
|  | *P. aeruginosa* | *30/30* | *🗴* | *🗸* | *4033* | (6.32) |  |
| C120 | *S. aureus* | *29* | *🗴* | *🗸* | *747* | (0.83) |  |
|  | *H. influenzae* | *20/21* | *🗴* | *🗸* | *57389* | (63.78) |  |
| C121 | *P. aeruginosa* | *20/20* | *🗸* | *🗸* | *67527* | (72.96) |  |
| C122 | *S. aureus* | *32/31* | *🗴* | *🗴* | *336* | (0.73) |  |
| C123 | *P. aeruginosa* | *32/31* | *🗴* | *🗸* | *3108* | (12.90) |  |
| ^N/A: insufficient sample available for sequencing. * Validation is reported as >500 reads and present in >0.5% of total classified reads^ | | | | | | | |

| **Study ID** | **TaqMan Array Card** | | | **Routine respiratory virus multiplex qPCR** | | **Concordance** |
| --- | --- | --- | --- | --- | --- | --- |
|  | **Sample method** | **Detection** | **Cycle threshold(s)** | **Sample method** | **Detection(s)** |  |
| C021 | Mini-BAL | *Rhinovirus* | 28/25 | Mini-BAL | *Adenovirus* | *🗴* |
| C028 | Mini-BAL | *-* |  | NPA | *Rhinovirus* | *🗴* |
| C029 | Mini-BAL | *-* |  | ETT aspirate | *-* | *🗸* |
| C030 | ETT aspirate | *-* |  | ETT aspirate | *-* | *🗸* |
| C032 | Mini-BAL | *Rhinovirus* | 23/25 | ETT aspirate | *Rhinovirus* | *🗸* |
| C034 | Mini-BAL | *-* |  | NPA | *-* | *🗸* |
| C035 | Mini-BAL | *Rhinovirus* | 26 | ETT aspirate | *Rhinovirus*  *(+ Enterovirus)* | *🗸* |
| C036 | Mini-BAL | *Rhinovirus* | 23 | ETT aspirate | *Rhinovirus* | *🗸* |
| C040 | Mini-BAL | *-* |  | NPA | *Rhinovirus*  *(+ Enterovirus)* | *🗴* |
| C045 | Mini-BAL | *-* |  | NPA | - | *🗸* |
| C049 | Mini-BAL | *-* |  | OP swab | - | *🗸* |
| C050 | Mini-BAL | *-* |  | Mini-BAL | - | *🗸* |
| C053 | Mini-BAL | *Rhinovirus* | 21/21 | NPA | *Picornavirus* | *🗸* |
| C054 | Mini-BAL | *Rhinovirus* | 22/20 | NPA | *Picornavirus* | *🗸* |
| C058 | Mini-BAL | *-* |  | NPA | *Rhinovirus* | *🗴* |
| C068 | Mini-BAL | *Human parainfluenza virus 3* | 18/19 | NPA | *Human parainfluenza virus 3* | *🗸* |
| C077 | Mini-BAL | *Bocavirus* | 17 | NPA | *-* | *🗴** |
| C083 | Mini-BAL | *RSV* | 20/23 | NPA | *RSV* | *🗸* |
| C085 | Mini-BAL | *Rhinovirus* | 25/24 | NPA | *Rhinovirus* | *🗸* |
| C087 | Mini-BAL | *Rhinovirus* | 20/21 | NPA | *Rhinovirus* | *🗸* |
| C088 | Mini-BAL | *-* |  | NPA | *-* | *🗸* |
| C089 | Mini-BAL | *Rhinovirus* | 24/21 | NPA | *Rhinovirus* | *🗸* |
| C097 | Mini-BAL | *Rhinovirus* | 22/22 | NPA | *Rhinovirus* | *🗸* |
| C100 | Mini-BAL | *RSV* | 25/28 | OP/NP combined | *RSV* | *🗴* |
|  |  |  |  |  | *Coronavirus OC43* |  |
| C102 | Mini-BAL | *RSV* | 21/22 | OP/NP combined | *RSV* | *🗸* |
| C103 | Mini-BAL | *Enterovirus* | 18/22 | OP/NP combined | *Adenovirus*  *(+ Picornavirus)* | *🗴* |
| C108 | Mini-BAL | *Rhinovirus* | 23/25 | OP/NP combined | *Parainfluenza virus 4*  *(+ Picornavirus)* | *🗴* |
| C109 | Mini-BAL | *Rhinovirus* | 28/27 | OP/NP combined | *Picornavirus* | *🗸* |
| C110 | Mini-BAL | *-* |  | NP swab | *-* | *🗸* |
| C120 | Mini-BAL | *-* |  | OP/NP combined | *-* | *🗸* |
| C124 | Mini-BAL | *-* |  | OP/NP combined | *Picornavirus* | *🗴* |
| ^* Note that bocavirus is not included on routine viral respiratory multiplex tests in this institution, hence would not be expected to be identified on this sample. ETT: Endotracheal tube; Mini-BAL: non-bronchoscopic bronchoalveolar lavage; NP: Nasopharyngeal; NPA: Nasopharyngeal aspirate; OP: Oropharyngeal; RSV:^ *^Respiratory syncytial virus^* | | | | | | |

**Table S5** Detections of viruses on TaqMan Array Card (TAC) compared to samples obtained within 24 hours that underwent respiratory viral multiplex testing.

| **Characteristic** | **Control cohort**  N = 52 | **RASCALS cohort**  N = 100 | **p-value** |
| --- | --- | --- | --- |
| Age (years), median (IQR) | 0.6 (0.1 – 2.6) | 1.2 (0.3-5.2) | 0.029^a^ |
| Sex (male), n (%) | 34 (65) | 58 (58) | 0.377^b^ |
| Weight (kilograms), median (IQR) | 5.9 (3.9 – 13.5) | 10.6 (5.3 – 19.7) | 0.026^a^ |
| Weight-for-age Z score^$^, mean (SD) | -0.7 (1.5) | -0.2 (1.3) | 0.034^c^ |
| Suspected VAP, n (%) | 3 (6) | 20 (20) | 0.020^b^ |
| Need for vasoactive support, n (%) | 15 (29) | 32 (32) | 0.690^b^ |
| Days free of PICU at 28 days, mean (SD) | 18.5 (7.2) | 16.8 (7.9) | 0.216^c^ |
| Days free of invasive ventilation at 28 days, mean (SD) | 18.9 (8.8) | 19.1 (7.2) | 0.878^c^ |
| Survival to hospital discharge, n (%) | 49 (94) | 94 (94) | 0.954^b^ |
| PIM3 score, median (IQR) | 0.8 (0.4 – 4.2) | 3.2 (0.5 – 5.0) | 0.029^a^ |
| ^a = Mann-Whitney U test; b = Chi-square test for independence; c = Student’s t-test for equality of means, two-sided p, equal variance not assumed; IQR = Interquartile range; LRTI = Lower respiratory tract infection; PIM3 = Paediatric Index of Mortality 3[10]; SD = Standard deviation; VAP = Ventilator associated pneumonia $ = Determined by based on growth charts produced by the World Health Organisation in children aged <2 years with correction for prematurity, and Centres for Disease Control and Prevention in children aged >2 years.^ | | | |

**Table S6** Demographics of children in control cohort versus the RASCALS cohort

**Table S7** Demographics of children with suspected community acquired pneumonia

| **Characteristic** | **Control cohort**  N = 49 | **RASCALS cohort**  N = 80 | **p-value** |
| --- | --- | --- | --- |
| Age (years), median (IQR) | 0.6 (0.1 – 2.6) | 1.1 (0.3 – 4.8) | 0.101^a^ |
| Sex (male), n (%) | 33 (67) | 44 (55) | 0.165^b^ |
| Weight (kilograms), median (IQR) | 6.0 (5.0 – 18.5) | 9.8 (5.0 – 18.5) | 0.128^a^ |
| Weight-for-age Z score^$^, mean (SD) | -0.7 (1.5) | -0.2 (1.3) | 0.100^c^ |
| Need for vasoactive support, n (%) | 15 (31) | 23 (29) | 0.822^b^ |
| Days free of PICU at 28 days, mean (SD) | 19.2 (6.7) | 18.0 (7.7) | 0.347^c^ |
| Days free of invasive ventilation at 28 days, mean (SD) | 20.0 (7.0) | 20.1 (6.5) | 0.911^c^ |
| Survival to hospital discharge, n (%) | 46 (94) | 77 (96) | 0.535^b^ |
| PIM3 score, median (IQR) | 0.6 (0.1 – 2.5) | 1.1 (0.3 – 4.7) | 0.153^a^ |
| Days on antimicrobial therapy, mean % (SD) | 65.5 (29.3) | 75.9 (33.0) | 0.065^c^ |
| ^a = Mann-Whitney U test; b = Chi-square test for independence; c = Student’s t-test for equality of means, two-sided p, equal variance not assumed; IQR = Interquartile range; LRTI = Lower respiratory tract infection; PIM3 = Paediatric Index of Mortality 3[10]; SD = Standard deviation; VAP = Ventilator associated pneumonia. $ = Determined by based on growth charts produced by the World Health Organisation in children aged <2 years with correction for prematurity, and Centres for Disease Control and Prevention in children aged >2 years.^ | | | |

**Table S8** Comparison of antimicrobial use by class in children with suspected community acquired pneumonia

| **Antimicrobial Class** | **Control cohort**  N = 49  PICU days = 430 | | **RASCALS cohort**  N = 80  PICU days = 766 | | **p-value*** |
| --- | --- | --- | --- | --- | --- |
|  | Treatment days | % | Treatment days | % |  |
| Aminoglycoside | 22 | 5.1 | 20 | 2.6 | 0.024 |
| Beta-lactam | 234 | 54.4 | 470 | 61.4 | 0.019 |
| Fluoroquinolone | 10 | 2.3 | 9 | 1.2 | 0.127 |
| Glycopeptide | 7 | 1.6 | 25 | 3.3 | 0.093 |
| Macrolide | 94 | 21.9 | 127 | 16.6 | 0.024 |
| Other | 28 | 6.5 | 33 | 4.3 | 0.096 |
| ^PICU: Paediatric intensive care unit; *Chi square test^ | | | | | |

|  |
| --- |

**References**

1. Magoc T, Salzberg S (2011) FLASH: Fast Length Adjustment of Short Reads to Improve Genome Assemblies. Bioinformatics 27:2957–2963. https://doi.org/10.1093/bioinformatics/btr507

2. Bokulich NA, Subramanian S, Faith JJ, et al (2013) Quality-filtering vastly improves diversity estimates from Illumina amplicon sequencing. Nat Methods 10:57–59. https://doi.org/10.1038/nmeth.2276

3. Caporaso JG, Kuczynski J, Stombaugh J, et al (2010) QIIME allows analysis of high-throughput community sequencing data. Nat Methods 7:335–336. https://doi.org/10.1038/nmeth.f.303

4. Edgar RC (2013) UPARSE: highly accurate OTU sequences from microbial amplicon reads. Nat Methods 10:996–998. https://doi.org/10.1038/nmeth.2604

5. Altschul SF, Gish W, Miller W, et al (1990) Basic local alignment search tool. J Mol Biol 215:403–410. https://doi.org/https://doi.org/10.1016/S0022-2836(05)80360-2

6. Qiong W, M GG, M TJ, R CJ (2007) Naïve Bayesian Classifier for Rapid Assignment of rRNA Sequences into the New Bacterial Taxonomy. Appl Environ Microbiol 73:5261–5267. https://doi.org/10.1128/AEM.00062-07

7. Quast C, Pruesse E, Yilmaz P, et al (2013) The SILVA ribosomal RNA gene database project: improved data processing and web-based tools. Nucleic Acids Res 41:D590–D596. https://doi.org/10.1093/nar/gks1219

8. Salter SJ, Cox MJ, Turek EM, et al (2014) Reagent and laboratory contamination can critically impact sequence-based microbiome analyses. BMC Biol 12:87. https://doi.org/10.1186/s12915-014-0087-z

9. Gerber JS, Hersh AL, Kronman MP, et al (2017) Development and Application of an Antibiotic Spectrum Index for Benchmarking Antibiotic Selection Patterns Across Hospitals. Infect Control Hosp Epidemiol 38:993–997. https://doi.org/DOI: 10.1017/ice.2017.94

10. Straney L, Clements A, Parslow RC, et al (2013) Paediatric index of mortality 3: an updated model for predicting mortality in pediatric intensive care*. Pediatr Crit Care Med 14:673–681. https://doi.org/10.1097/PCC.0b013e31829760cf
